# Racial discrimination is associated with binge-eating disorder in early adolescents: a cross-sectional analysis

**DOI:** 10.1186/s40337-023-00866-0

**Published:** 2023-08-17

**Authors:** Julia H. Raney, Abubakr A. Al-Shoaibi, Iris Y. Shao, Kyle T. Ganson, Alexander Testa, Dylan B. Jackson, Jinbo He, David V. Glidden, Jason M. Nagata

**Affiliations:** 1 Department of Pediatrics, Division of Adolescent and Young Adult Medicine, University of California, San Francisco, CA USA; 2 Factor-Inwentash Faculty of Social Work, University of Toronto, Toronto, ON Canada; 3 Department of Management, Policy and Community Health, University of Texas Health Science Center at Houston, Houston, TX USA; 4 Department of Population, Family and Reproductive Health, Johns Hopkins Bloomberg School of Public Health, Baltimore, MD USA; 5 School of Humanities and Social Science, The Chinese University of Hong Kong, Shenzhen, Guangdong, 518172 China; 6 Department of Epidemiology and Biostatistics, University of California, San Francisco, CA USA

**Keywords:** Racial discrimination, Binge-eating disorder, Adolescent health

## Abstract

**Background:**

Racial and ethnic discrimination are known stressors and are associated with negative psychological and physical health outcomes. Previous studies have found relationships between racial/ethnic discrimination and binge-eating disorder (BED), though they have mainly focused on adult populations. The aim of this study was to determine associations between racial/ethnic discrimination and BED in a large, national cohort study of early adolescents. We further sought to explore associations between the racial/ethnic discrimination perpetrator (students, teachers, or other adults) and BED.

**Methods:**

We analyzed cross-sectional data from the Adolescent Brain Cognitive Development Study (N = 11,075, 2018–2020). Logistic regression analyses examined associations between self-reported experiences of racial or ethnic discrimination and binge-eating behaviors and diagnosis, adjusting for potential confounders. Racial/ethnic discrimination measures were assessed based on the Perceived Discrimination Scale, which measures experiences of discrimination based on race/ethnicity and frequency of ethnic discrimination by teachers, adults outside of school, and students. Binge-eating behaviors and diagnosis were based on the Kiddie Schedule for Affective Disorders and Schizophrenia (KSAD-5).

**Results:**

In this racially diverse sample of adolescents (N = 11,075, age range 9–12 years), 4.7% of adolescents reported racial or ethnic discrimination and 1.1% met the criteria for BED. In the adjusted models, racial/ethnic discrimination was associated with 3 times higher odds of having BED (OR 3.31, CI 1.66–7.74). Further, experiences of ethnic discrimination by students and adults outside school were associated with significantly increased odds of BED diagnosis (OR 1.36, CI 1.10–1.68 and OR 1.42 CI 1.06–1.90, respectively)., Increased odds of binge eating behaviors were only significantly associated with ethnic discrimination perpetuated by students (OR 1.12, CI 1.02–1.23).

**Conclusions:**

Children and adolescents who have experienced racial/ethnic discrimination, particularly when discrimination was perpetuated by other students, have higher odds of having binge-eating behaviors and diagnoses. Clinicians may consider screening for racial discrimination and providing anti-racist, trauma-informed care when evaluating and treating patients for BED.

**Supplementary Information:**

The online version contains supplementary material available at 10.1186/s40337-023-00866-0.

## Abstract (plain language summary)

Binge-eating disorder is associated with significant psychological and physical consequences including depression, anxiety, impaired relationships, and increased cardiometabolic risks. Recent research has demonstrated that many of binge-eating behaviors develop in early adolescence, a time of immense psychosocial development. Racial and ethnic discrimination are known stressors, and previous studies have found relationships between racial and ethnic discrimination and binge-eating disorder, though they have mainly focused on adult populations. This study helps fill that gap by using data from the Adolescent Brain Cognitive Development Study Study, the largest prospective study of adolescent brain development in the US. In this large, racially diverse, national study of 11,075 adolescents aged primary 10–11 years old, we find that early adolescents who have experienced racial/ethnic discrimination, particularly when discrimination was perpetuated by other students, have higher odds of having binge-eating behaviors and diagnoses. These findings have important school and clinical implications. For example, schools may consider implementing curricula focused on anti-racist practices that foster environments where all youth to thrive. In addition, we recommend that clinicians screen for racial discrimination and provide culturally sensitive, equity-focused care when evaluating and treating patients with binge-eating disorder.

**Supplementary Information:**

The online version contains supplementary material available at 10.1186/s40337-023-00866-0.

## Background

Binge-eating disorder (BED), characterized by consuming a large amount of food while feeling a loss of control and negative emotions [1], is estimated to affect 4.5% of the population across the lifetime [2], with those from minoritized backgrounds experience even higher rates of BED and binge-eating behaviors [3, 4]. BED is associated with significant psychological and physical consequences including depression, anxiety, impaired relationships, and increased cardiometabolic risks [2, 5, 6]. Given BED’s prevalence, consequences, and challenges with accessing treatment, identifying risk factors for the development of BED is critical to promote health and health equity.

Racial discrimination, or personally mediated racism, has been recognized as a core driver of health inequities in adolescents and children [7–9]. Personally mediated racism includes experiences of stereotypes and prejudices about a person’s ability, intent, or motives on the basis of race [10]. Personally mediated racism can be expressed implicitly or explicitly and can be experienced directly or indirectly. Minoritized children and adolescents face personally mediated racism in their interactions with teachers and students at school, during extracurricular activities, and increasingly in online, digital environments [11, 12]. Experiencing racial harassment and taunts can over-activate the stress response, and have cascading effects including increased and prolonged levels of exposure to stress hormones and oxidative stress [13]. A growing body of literature has found associations between racial discrimination and BED. Several studies of Latino and African American adults have demonstrated significant associations between racial discrimination and binge eating [14–16]. For example, a study of a nationally representative sample of Latino individuals found a significant association with discrimination and binge eating, with the average age in the sample being 40 years old (range 18–97) [15]. In addition, Assari et al.’s paper demonstrated significant associations between perceived discrimination and BED in a nationally representative example of African American adults (average age 42) [16]. Studies have also found significant associations between binge eating behaviors in young adult Black women (ages 18–25) [17] and Latino young adults (ages 18–25) [18].

Several theoretical models have been proposed to explain this relationship between racial/ethnic discrimination and BED. The minority stress model theorizes that minorized groups experience unique psychosocial stressors that elevate risk for mental health problems including binge-eating [19, 20]. Another possible mechanism is the affect regulation model, where binge-eating episodes are triggered by negative affect (e.g., depression, anxiety) in an effort to decrease the negative affect [21, 22]. Given that racial discrimination is associated with depression and anxiety, people who experience discrimination may be at greater risk for BED through this pathway [23–25]. A third potential mechanism is that minoritized racial and ethnic groups having increased risks of experiencing racial/ethnic discrimination [26, 27] and food insecurity, which may result in an increased risk of BED among people who have experienced discrimination [28].

However, little is known about the association between racial discrimination and the development of BED in early adolescents. A better understanding of the development of BED in this age-group is especially critical for several reasons. First, recent research has demonstrated that binge eating behaviors develop in early adolescents, a time of immense psychosocial development [29]. A study of 10- to 11-year old children from the ABCD Study, a large, diverse, population-based sample, estimated the prevalence of BED to be 1.1% [30]. In addition, a population-based study of 14 year old early adolescents found a 14% prevalence rate of subclinical binge-eating behaviors [31]. Second, early adolescents experience racism and discrimination at unacceptable rates; a recent study estimated that 4.8% of 10- to 11- year old children reported being treated unfairly because of their race, ethnicity or color, and 10% of Black children reported experiencing racism [32]. Given the prevalence of perceived discrimination experienced by youth in this age group, it is critical to characterize the public health effects and rapidly implement antiracism practices. Given the significant impact that peers, teachers, and non-caregiver adults have on early adolescent development [33–35], we further sought to characterize the impacts of expressed by these varying groups.

The purpose of the current study was to examine the associations between racial discrimination and BED among a large, diverse cohort of early-adolescents ages 9–12 years old (primarily ages 10–11). We hypothesized that perceived racial discrimination would be associated with binge eating behaviors and diagnoses.

## Methods

### Study population

This study uses survey data from the Adolescent Brain Cognitive Development (ABCD) Study to determine the association between racial discrimination and BED among US early adolescents. The ABCD Study is a large, prospective cohort study of brain development, health, and health behaviors among US adolescents across 21 recruitment sites [36]. The 21 recruitment sites are listed in the appendix (Supplemental Appendix). The ABCD study was designed to reflect the sociodemographic diversity of the United States. Recruitment was primarily school-based, though also included mailing lists, referrals, and outreach to summer activities such as camps as the Boys and Girls Clubs, YMCA’s, and summer meals programs. To prevent higher attrition rates from low-income families, during research visits, the study provides a free nutrition and exercise program, a meal, homework assistance, childcare for other family members who attended the participant to the visits, and transportation vouchers as needed [23]. We included data collected between 2018 and 2020, corresponding to Year one of the ABCD Study, the first year adolescent-reported racial discrimination was assessed. Participants missing data for (1) sociodemographic characteristics or (2) all discrimination questions (n = 710) were excluded, yielding the total sample of 11,075. All participants gave assent, and parents/caregivers provided signed informed consent. The ABCD Study protocol was approved by the Institutional Review Board of the University of California, San Diego and at each respective study site.

### Exposure: racial discrimination

Racial discrimination was measured using the Perceived Discrimination Scale [23, 37], which was developed to measure adolescents' perception of being unaccepted in society or being unwanted based on their racial or ethnic background or skin color. Adolescents were asked, “In the past 12 months, have you felt discriminated against: because of your race, ethnicity, or color?” In addition, adolescents were asked how often they had been treated unfairly or negatively because of their ethnic background by each of the following groups: teachers, adults outside the school, and students (1 = almost never; 2 = rarely; 3 = sometimes; 4 = often; 5 = very often).

### Outcome: binge-eating disorder

BED diagnosis and behaviors were assessed that the one-year follow-up through parent/caregiver responses to the Kiddie Schedule for Affective Disorders and Schizophrenia (KSADS-5), a computerized tool developed to categorize child and adolescent mental health based on the DSM-5 [1, 38]. Parents/caregivers completed all modules of the KSADS-5 to characteristics, frequency, and duration of their child’s binge-eating behaviors as well as associated distress. The presence of binge-eating behaviors was assessed by asking the caregiver if their child experienced a loss of control of their eating and ate way more than he/she needed in the last two weeks. If the caregiver reported this had happened in the last two weeks, the adolescent was determined to have binge- eating behaviors. BED was determined using the KSADS-5 computerized scoring system, where responses to the survey questions were extrapolated into their respective diagnosis based on reported behaviors corresponding to the DSM-5. Although bulimia nervosa (BN) also consists of binge eating symptoms, the prevalence of BN in the sample was low (6 adolescents, 0.05% of the study population) and therefore this study focused on binge-eating behaviors and BED. Of note, all 6 adolescents diagnosed with BN were found to have binge-eating behaviors but zero of these adolescents were diagnosed with BED. These six participants were included in the binge-eating behaviors analysis because of (1) the low prevalence rate of BN in this sample and (2) the fact that binge eating behaviors are present in youth with BN and risk factors for the development of BN and binge-eating behaviors likely overlap. No youth in this sample were diagnosed with anorexia nervosa binge purge subtype (AN-BP).

### Covariates

We selected potential confounders for the association between racial discrimination and BED based on prior literature and theory [16, 39]. Age, sex (male, female), race/ethnicity (White, Latino/Hispanic, Black, Asian, Native American, Other), nativity (youth born in US or outside of the US), household income ($24,999 or less, $25,000 through $49,999, $50,000 through $74,999, $75,000 through $99,999, $100,000 through $199,999, $200,000 and greater), and highest parent education (high school or less vs. college or more) were selected from parent self-report data at baseline. ABCD Study site ([21] total sites)was also included to adjust for potential regional variation.

### Statistical analyses

Differences in discrimination scales by group were estimated with an ANOVA. Unadjusted and adjusted logistic regressions were conducted using Stata 17.0 (StataCorp, College Station, TX) to estimate associations between past year experiences of racial/ethnic discrimination and BED diagnosis and behaviors. In addition, unadjusted and adjusted logistic regression analyses estimated the association between frequency of ethnic discrimination (teachers, adults outside school, students), and BED diagnosis and behaviors. To independently assess for the effect of race/ethnicity, we included three models. Model A includes no confounding, Model B includes all covariates except for race/ethnicity, and Model C includes all covariates. The ABCD study sociodemographic variables were standardized to match the distribution American Community Survey from the U.S. Census [40].

## Results

The 11,075 adolescent respondents were racially and ethnically diverse (standardized percentages: 53.4% White, 19.6% Latino/Hispanic, 16.5% Black, 5.6% Asian, 3.2% Native American, 1.4% Other, Table 1). Binge-eating behaviors and diagnosis were present at 7.9% (791) and 1.1% (n = 105), respectively. Approximately one in twenty youth reported experiencing racial or ethnic discrimination in the past year. In addition, there was a statistically significant difference in discrimination scorse across groups; adolescents reported that teachers, adults outside of school, and students perpetuated ethnic discrimination at 8.0%, 9.8%, and 25.1%, respectively (F statistic 630, *p* value < 0.001).

**Table 1:** Sociodemographic characteristics of participants in the Adolescent Brain Cognitive Development Study (ABCD) Study, 2018–2020, (n = 11,075)

	Mean (SD) or %
*Demographic characteristics*	
Age	11.0 (0.6)
Sex	
Female	48.8%
Male	51.2%
Race/ethnicity	
White	53.4%
Latino/Hispanic	19.6%
Black	16.5%
Asian	5.6%
Native American	3.2%
Other	1.4%
Nativity	
Youth born in U.S	96.3%
Youth born outside U.S	3.7%
Highest parental education	
High school education or less	15.6%
College education or more	84.4%
Household income	
$24,999 or less	16.9%
$25,000 to $49,999	20.2%
$50,000 to $74,999	18.2%
$75,000 to $99,999	16.0%
$100,000 to $199,999	21.7%
$200,000 and greater	7.0%
*Type of discrimination reported*	
Discrimination because of race, ethnicity, or color	4.7%
*Been treated unfairly or negatively because of your ethnic background by*	
Teachers	
Almost never	92.0%
Rarely	4.3%
Sometimes	2.1%
Often	0.9%
Very Often	0.7%
Adults outside school	
Almost never	90.2%
Rarely	6.5%
Sometimes	2.2%
Often	0.6%
Very Often	0.5%
Students	
Almost never	74.9%
Rarely	13.1%
Sometimes	7.8%
Often	2.2%
Very Often	2.0%
Binge eating	
Binge-eating behaviors	7.9%
Binge-eating disorder diagnosis	1.1%

ABCD Study sociodemographic variables were standardized to match the distribution American Community Survey from the U.S. Census

In both the adjusted and unadjusted models, racial/ethnic discrimination was associated with increased binge-eating behaviors and binge-eating disorder diagnosis (Table 2). In Panel C, adolescents who reported perceived discrimination had 3.31 higher odds of BED (95% CI 1.66–6.63). Increased frequency of ethnic discrimination by students was also significantly associated with a higher odds of BED diagnosis and behaviors. In addition, respondents who reported more frequent ethnic discrimination by adults outside of school had significantly higher odds of BED diagnosis.

**Table 2:** Associations between discrimination and binge-eating behaviors and diagnosis (N = 11,075)

	Binge-eating behaviors	Binge-eating disorder diagnosis
	OR (95% CI)	OR (95% CI)
*Panel A: bivariate model*		
Discrimination because of race, ethnicity, or color	2.22***	4.31***
	(1.62–3.05)	(2.40–7.74)
Been treated unfairly or negatively because of your ethnic background by:		
Teachers	1.20**	1.44**
	(1.06–1.36)	(1.16–1.79)
Adults outside school	1.19*	1.55**
	(1.03–1.37)	(1.19–2.01)
Students	1.18***	1.54***
	(1.08–1.28)	(1.28–1.86)
*Panel B: with confounding variables except race/ethnicity*		
Discrimination because of race, ethnicity, or color	2.10***	3.27**
	(1.49–2.96)	(1.67–6.41)
Been treated unfairly or negatively because of your ethnic background by:		
Teachers	1.12	1.26
	(0.98–1.29)	(0.99–1.61)
Adults outside school	1.11	1.44*
	(0.95–1.30)	(1.08–1.93)
Students	1.12*	1.36**
	(1.02–1.23)	(1.10–1.68)
*Panel C: with all confounding variables*		
Discrimination because of race, ethnicity, or color	2.12***	3.29*
	(1.50–3.00)	(1.65–6.57)
Been treated unfairly or negatively because of your ethnic background by:		
Teachers	1.12	1.25
	(0.97–1.28)	(0.98–1.60)
Adults outside school	1.11	1.42*
	(0.95–1.30)	(1.06–1.90)
Students	1.12*	1.36**
	(1.02–1.23)	(1.10–1.68)

*Indicates *p* < 0.05, **indicates *p* < 0.01, ***indicates significant at < 0.001. ABCD Study sociodemographic variables were standardized to match the distribution American Community Survey from the U.S. Census. Panel B models include sex, household income, parent education, and site. Panel C models include race/ethnicity, sex, household income, parent education, and site

## Discussion

In this national, sociodemographically diverse sample of early adolescents in the U.S., we found that 1.1% of adolescents had a BED diagnosis, which is similar to prior studies with adolescent prevalence estimates of 1.3–1.6% [6, 41]. Further, this study identified experiencing racial/ethnic discrimination was associated with greater odds of binge-eating behaviors and diagnosis, even when adjusting for confounding factors including race, sex, nativity, parental education, and socioeconomic status.

The relationship between discrimination and binge-eating is consistent with prior studies in minoritized adult populations that have demonstrated associations between experiencing racial/ethnic discrimination and binge-eating behaviors in Latino and Black the general adult population and young adults [14, 16]. Our findings contribute to the literature by demonstrating that perceived discrimination is significantly associated with higher odds of binge-eating behaviors and diagnosis in a national, diverse population of US early adolescents; importantly, early-adolescents represent an under-researched age group whose developmental period is vulnerable to developing health-related risk behaviors [29, 42]. As BED is associated with significant distress, morbidity, and mortality, it is critical to investigate risk factors in this age group to design primary and secondary prevention interventions [43, 44].

These findings are consistent with numerous theoretical perspectives including the minority stress model and the affect regulation model [19–22]. Similarly, the findings are also consistent with prior literature on the inequitable distribution if sociodemographic risk factors across race/ethnicity (ie food insecurity), which places individuals at higher risk for BED [26–28]. To further explore these theories, future studies should explore whether these factors (e.g., anxiety, stress, depression, food insecurity) mediate the associations between racial discrimination and BED.

Our study further adds to the literature by exploring how unique groups of perpetrators influence the association between discrimination and BED. In our study, adolescents reported students to be the most common perpetuators of ethnic discrimination with one if four adolescents reporting experiencing ethnic discrimination by students rarely or more frequently; in addition, reporting ethnic discrimination perpetuated by students was significantly associated with increased odds of binge-eating disorder behaviors and diagnosis. The significant impact of peer discrimination on adolescent’s mental health has been supported in prior literature [34, 45]. From a developmental perspective, peer discrimination may be particularly impactful for early adolescents as they increasing spend time outside of the home and rely more on peers for psychosocial acceptance, self-concept, socialization, and identity formation [46, 47]. Several studies have shown that peer victimization in early adolescence is predictive of subsequent development of depressive symptoms [48, 49]. Our study builds upon these studies by highlighting that discrimination is also associated with BED in a national, diverse sample of early adolescents. Of note, experiencing discrimination by other adults outside school was also associated with a significantly higher odds of BED diagnosis. This is consistent with literature that shows the important influence that nonparental adults, such as mentors and police, can have on adolescent mental health [50–52].

Importantly, in the adjusted models, discrimination perpetuated by teachers was not significantly associated with greater odds of binge-eating behaviors or diagnosis and discrimination by adults outside school was not associated with binge eating behaviors. However, this may be partially due to the fewer cases of discrimination from teachers. Prior studies have found that teachers play a critical role in adolescent development and mental health [53, 54]. Further studies should continue to investigate the relationships between ethnic discrimination perpetuated by teachers and adults outside school and binge eating.

This study has several limitations. First, the Perceived Discrimination Scale asked about ethnic discrimination by other students, not peers, so this may have missed perpetuators who they interacted with outside of school. Additionally, this study used parent report of binge eating diagnosis and behaviors. While parents are important reporters for eating disorders in early adolescents as children have less insight into their eating behaviors [36, 55], parent and child reports of binge-eating behaviors have a tendency of low concordance with parents reporting higher rates of binge eating [36, 55, 56]. This may have caused overestimation of BED in this study, though more studies are needed to determine if parent or child report of symptoms is most accurate.

Another concerns is that this manuscript’s analyses of racial/ethnic discrimination adjusted for several sociodemographic correlates including race/ethnicity, which is a socially constructed categorization that serves as a proxy for exposure to systemic racism and racial discrimination [57, 58]. This may make the study vulnerable to underestimating the associations between racial/ethnic discrimination and BED. Even so, race/ethnicity was included as a control variable to reduce the likelihood of spurious results, and Panel B, which omitted statistical controls for race/ethnicity, yielded substantively similar results. In our findings, the associations between racial/ethnic discrimination and BED were significant across all models. In addition, this study assesses reported experiences or racism over the past year but does not explore the cumulative impact of racism or the experience of youth with intersectional identities; we recommend that future studies employ an intersectional lens that explores the associations the impact of discrimination on BED among those with multiple marginalized identities (e.g. sexual minority Latinx adolescents), considering that these youth are at risk for unique types of discrimination [59]. Further, while the ABCD study includes adolescents from many diverse backgrounds, the study population’s parents are more highly educated and has a greater percentage of white children (53% vs. 50%) and a lower percentage of Latinx children (20 vs. 26%) compared to the 2020 US Census Bureau [60]; accordingly, the study may underestimate the full impact of racial discrimination on US youth from lower education background and Latinx families. Lastly, this study is cross-sectional, and does not assess the longitudinal impact of perceived racism. We recommend that future studies explore this relationship.

## Conclusions

This study demonstrates that experiencing racial or ethnic discrimination in early adolescence is associated with greater odds of BED diagnosis and symptoms, which has important school, public policy, and clinical implications. For example, schools may consider implementing curricula focused on anti-racist practices that foster environments where all youth to thrive [61]. In addition, minoritized populations, for instance, have historically received inadequate access to eating disorder care and inclusion in eating disorders research, which increases the risk of delayed and poorer outcomes [62] Policy changes that that target these systemic issues, such as increased education about BED among diverse populations and increased access to eating disorder trained mental health professionals [62], may profoundly impact the risk and treatment outcomes of BED.

The US Preventive Services Task Force (USPSTF) recently reviewed eating disorder screening in asymptomatic adolescents and adults and determined there to be insufficient evidence to recommend routine screening in this population, especially among racial/ethnic minority populations [63]. However, clinicians may still consider screening for eating disorder behaviors in early adolescents with significant risk factors, such as racial discrimination, given the significant physician and mental health consequences of eating disorders [64]. If experiences of racism are identified, we recommend assessing families for social determinants of health often associated with racism (e.g., food insecurity, housing inequity) to connect families to resources [65, 66]. Broadly, clinicians may integrate positive youth development approaches including racial socialization, to identify strengths and youth and family protective factors that can help mitigate exposure to racist behaviors use [65, 67]. Second, clinicians may implement strategies such as the Raising Resisters approach, which recognizes the various forms of racism, differentiates racism from other unfair treatment, opposes the negative messages, and replaces those messages with something positive [68]. Further research is needed to identify eating disorder treatment strategies that effectively provide coping strategies to manage racial/ethnic discrimination [15].

### Supplementary Information

**Additional file 1. **Supplemental Appendix.

## Data Availability

Data used in the preparation of this article were obtained from the ABCD Study (https://abcdstudy.org), held in the NIMH Data Archive (NDA). Investigators can apply for data access through the NDA (https://nda.nih.gov/).

## Abbreviations

ACEs: Adverse childhood experiences
ABCD study: Adolescent Brain Cognitive Development
AN-BP: Anorexia nervosa binge purge subtype
BED: Binge-eating disorder
KSADS-5: Kiddie Schedule for Affective Disorders and Schizophrenia
BN: Bulimia nervosa
SD: Standard deviation
