# Supplementary material for: Racial discrimination is associated with binge-eating disorder in early adolescents: a cross-sectional analysis
Source: J Eat Disord. 2023 Aug 17;11:139. doi: 10.1186/s40337-023-00866-0 (PMC10433622; doi:10.1186/s40337-023-00866-0)
Supplement: Supplementary file 1 — Additional file 1. Supplemental Appendix. [file 40337_2023_866_MOESM1_ESM.docx]

Supplemental Information

**21 Recruitment Sites Across the US**

• Children’s Hospital Los Angeles, Los Angeles, California

• Florida International University, Miami, Florida

• Laureate Institute for Brain Research, Tulsa, Oklahoma

• Medical University of South Carolina, Charleston, South Carolina

• Oregon Health and Science University, Portland, Oregon

• SRI International, Menlo Park, California

• University of California San Diego, San Diego, California

• University of California Los Angeles, Los Angeles, California

• University of Colorado Boulder, Boulder, Colorado

• University of Florida, Gainesville, Florida

• University of Maryland at Baltimore, Baltimore, Maryland

• University of Michigan, Ann Arbor, Michigan

• University of Minnesota, Minneapolis, Minnesota

• University of Pittsburgh, Pittsburgh, Pennsylvania

• University of Rochester, Rochester, New York

• University of Utah, Salt Lake City, Utah

• University of Vermont, Burlington, Vermont

• University of Wisconsin—Milwaukee, Milwaukee, Wisconsin

• Virginia Commonwealth University, Richmond, Virginia

• Washington University in St. Louis, St. Louis, Missouri

• Yale University, New Haven, Connecticut
